# Ultrafast Nucleic Acid Detection Equipment with Silicon-Based Microfluidic Chip

**DOI:** 10.3390/bios13020234

**Published:** 2023-02-07

**Authors:** Jiali Zhang, Zhuo Yang, Liying Liu, Tinglu Zhang, Lilei Hu, Chunrui Hu, Hu Chen, Ruihua Ding, Bo Liu, Chang Chen

**Affiliations:** 1 School of Microelectronics, Shanghai University, Shanghai 201800, China; 2 Shanghai Industrial Technology Research Institute (SITRI), Shanghai 201800, China; 3 Shanghai Si-Gene Biotechnology Co., Ltd., Shanghai 201800, China; 4 State Key Laboratory of Transducer Technology, Shanghai Institute of Microsystem and Information Technology, Chinese Academy of Sciences, Shanghai 200050, China; 5 Shanghai Academy of Experimental Medicine, Shanghai 200052, China

**Keywords:** nucleic acid amplification detection, polymerase chain reaction (PCR), microfluidic Chip, ultrafast PCR equipment

## Abstract

Recently, infectious diseases, such as COVID-19, monkeypox, and Ebola, are plaguing human beings. Rapid and accurate diagnosis methods are required to preclude the spread of diseases. In this paper, an ultrafast polymerase chain reaction (PCR) equipment is designed to detect virus. The equipment consists of a silicon-based PCR chip, a thermocycling module, an optical detection module, and a control module. Silicon-based chip, with its thermal and fluid design, is used to improve detection efficiency. A thermoelectric cooler (TEC), together with a computer-controlled proportional–integral–derivative (PID) controller, is applied to accelerate the thermal cycle. A maximum of four samples can be tested simultaneously on the chip. Two kinds of fluorescent molecules can be detected by optical detection module. The equipment can detect viruses with 40 PCR amplification cycles in 5 min. The equipment is portable, easily operated, and low equipment cost, which shows great potential in epidemic prevention.

## 1. Introduction

The outbreak of COVID-19 at the end of 2019 has caused substantial threat to public health. As of 14th Oct 2022, 6.2 billion cases have been confirmed leading to the death of more than 6.5 million people [1]. The economy of the world is negatively influenced by the ongoing epidemic [2]. To preclude the spread of the disease, rapid detection techniques are extensively required. PCR technology, with its high sensitivity and specificity, is helpful in the detection of infections [3]. The simple PCR detection can be conducted iteratively between different temperatures to achieve exponential amplification. Thus, a large number of detection equipment have been developed to detect viruses and infections through PCR technology [4,5,6].

The main drawback of conventional PCR is the long detection time (2–2.5 h) resulted from slow thermocycling. Ultrafast, portable diagnostic equipment are urgently demanded. Researchers have tried different ways to accelerate the thermocycling of PCR. Typical methods include using heaters with direct heating components like water bath [7,8], resistance heater [9,10], Peltier module [11,12], and indirect heating components like infrared ray heating [13,14], microwave heating [15,16], photon heating [17,18], and induction heating [19,20]. For PCR thermocycling forms, there are three main forms: static cavity PCR (SCPCR) [21], continuous flow PCR (CFPCR) [22,23] and convective PCR (CPCR) [24,25]. The SCPCR fixes the reagent in the chamber and realizes the PCR process by thermocycling module. The CFPCR divides the flow channel into different temperature regions, and the reagent continuously flows through different temperature regions to realize PCR thermocycling. CPCR heats the reagent at the end of the chamber. Due to the different density of fluid at different temperatures, the fluid spontaneously circulates through different temperature zones during the flow process.

The material of the chip also greatly affects the thermocycling efficiency. With a high thermal conductivity (120 W/(m·K)) [26], silicon shows great superiority over other materials like glass (1.5 W/(m·K)) [27] and plastic (0.2 W/(m·K)) [28] for fabrication of PCR microfluidic chips. The heating method, chip design and materials all affect the total detection time.

In this work, an ultrafast and portable PCR detection equipment is demonstrated. The equipment consists of TEC thermocycling module, silicon-based microfluidic chip, optical detection module, and control system. Since CFPCR cannot freely change the number of cycles, and the liquid flow of CPCR is instable, the SCPCR design is used here. TEC-based thermocycling system realizes ultrafast thermocycling. Silicon-based chip, with its high thermal conductivity, enhances the temperature uniformity of the tested sample. The optical detection module captures the fluorescence images in each cycle. After analyzing the fluorescence image, the fluorescence intensity curve can be read on the screen. The dimensions of the equipment are 228 mm × 205 mm × 151 mm (length × width × height), making it convenient to carry. The equipment can complete 40 PCR cycles in 5 min. Compared to the conventional PCR detection equipment, ultrafast equipment can shorten the detection time while ensuring the accuracy of detection.

## 2. Materials and Methods

### 2.1. Chip Design and Fabrication

The microfluidic silicon-based chip is designed and fabricated through wafer-scale semiconductor process.

**Figure 1:**
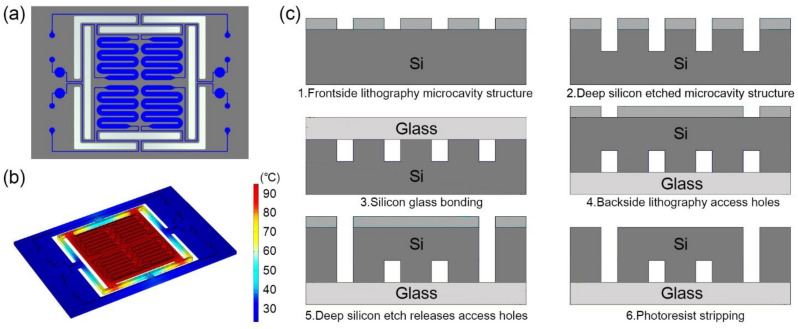
(**a**) Schematic diagram of the microfluidic chip. The chip is composed of four serpentine-shaped microchannels, and the buffer pool at the outlet can store the excess reagents. (**b**) Thermal simulation diagram when heating to 95 °C. The design of the insulation trench can make the main part of the chip maintaining temperature uniformity. (**c**) Schematic diagram of processing flow of microfluidic silicon-based chip.

Four serpentine-shaped microchannels are fabricated in one silicon-based microfluidic chip. The 17 mm × 25 mm rectangular microfluidic chip is designed with a volume of 6.5 μL for each serpentine-shaped microchannel, as shown in Figure 1a. The serpentine-shaped microchannels design can make full use of the limited space on the chip [29], and avoid bubbles during the injection process [4]. The bubble negatively influences the temperature stability during the PCR process. A circular buffer pool connected with an outlet opening is attached on each cavity to store the excess reagents.

The thermal insulation trench between the main part of the microfluidic chip and the margins is designed for the thermal insulation of the microchannels [30]. The insulation trench not only permits fast temperature shifting, but also avoids overheating the microfluidic chip and the parts that connected with it. The design enables each part of the serpentine-shaped microchannels to maintain the desired temperature when being heated, as shown in Figure 1b.

The fabrication process of the microfluidic chip includes standard semiconductor process. A bare glass substrate with the same size is anodic bonded to the microfluidic chip as a cover for the purpose of sealing the microchannels, as shown in Figure 1c.

### 2.2. PCR Kit and Thermocycling Protocol

The primers are the N gene primers of SARS-CoV-2 published by the Center for Disease Control (CDC), China. The positive sample is the pseudovirus containing the specific N gene fragment. The pseudovirus is wrapped with external nucleic acid fragment by MS2 phage shell protein, which is noninfectious, and has no autonomous replication ability. The pseudovirus contains sequence information as follows: N Gene SEQ GGCAGCAGTAGGGGAACTTCTCCTGCTAGAATGGCTGGCAATGGCGGTGATGCTGCTCTTGCTTTGCTGCTGCTTGACAGATTGAACCAGCTTGAGAGCAAAATGTCTGGTAAAGG. The PCR kit for ultrafast detection is KAP2G Fast HotStart PCR Kit (Roche, Basel, Switzerland). The PCR reaction has the final concentration of 1× buffer, 1 mM dNTP, 2.5 mM Mg^2+^, 0.36 μM the mixture of primer, 0.18 μM probe and 1U polymerase. The total volume is 5.5 μL. The ultrafast PCR detection for COVID N-gene procedure includes: 95 °C for 45 s; 40 temperature cycles of 95 °C for 0 s and 60 °C for 2 s. The qPCR probe method has higher specificity, and it does not need to observe the specificity through the melting curve like the dye method.

The PCR kit for the comparison with conventional equipment is Quantitative Diagnostic Kit for EBV-DNA (Sinomd, Bejing, China). The kit provides quantitative standards of 5 × 10^7^ copies/mL. Samples with concentrations of 5 × 10^6^ copies/mL, 5 × 10^5^ copies/mL, 5 × 10^4^ copies/mL are obtained by dilution. The conventional PCR detection reactions in this work are performed using SLAN-96P (HONGSHI, Shanghai, China). The PCR amplification procedure required by the kit includes: 95 °C for 3 mins; 40 temperature cycles of 94 °C for 15 s and 60 °C for 35 s.

### 2.3. Optical Detection Module

The miniaturized optical detection module includes blue/amber LED (CREE, Durham, NC, USA), collimating lens (BoYa, Shenzhen, China), blue/amber excitation filters (RuiCheng, Yixing, China), costumed dual-bandpass emission filter (RuiCheng, Yixing, China), CMOS camera (Sony, Tokyo, Japan), and corresponding structural parts.

**Figure 2:**
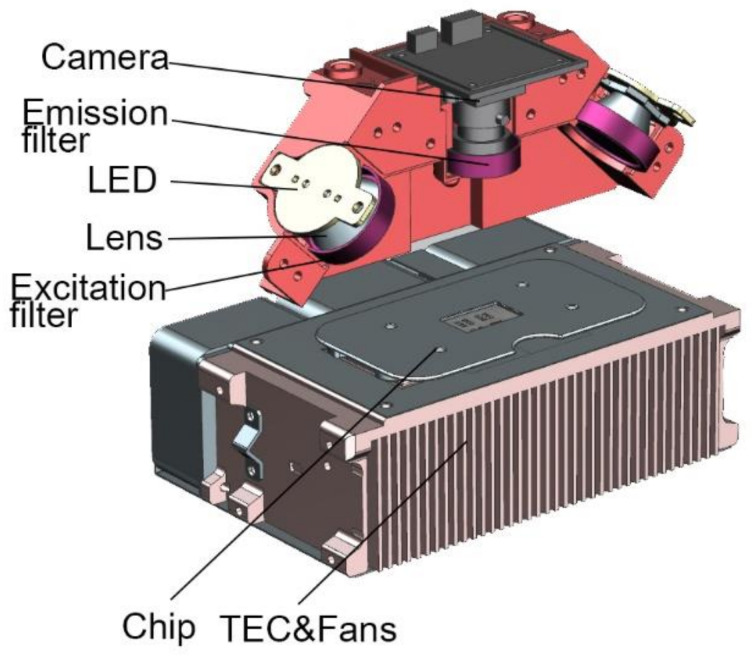
Optical detection system and thermocycling system.

Tiny blue and amber LED lamp beads are selected as the excitation light source. LED has characteristics of high luminous efficiency and fast response speed [31,32]. The working current is 0.2–0.7 A. The integrated LED lens is used as the collimation tool of the light source. FAM and ROX are chosen as fluorescent molecules. The light intensity of the excitation light source can be adjusted by changing magnitude of the current. The excitation light goes through excitation filter and the glass layer of the microfluidic chip before hitting the PCR mixture. The fluorescence emission light then passes through the emission filter to reach the CMOS camera, as shown in Figure 2.

### 2.4. Thermocycling Module

TEC (II-VI MARLOW, America) is applied to attain ultrafast and precise temperature control. The thermocycling system also includes a thermocouple (OMEGA, Norwalk, CT, USA), three fans (DELTA, Shanghai, China), and a customized heat sink, as shown in Figure 2.

TEC, based on Peltier effect, is installed under the silicon-based microfluidic chip for heating and cooling. TEC is ideal for the equipment due to its short response time and high temperature shifting rate [33]. A thermocouple is fixed close to the TEC heat sink to measure the temperature of the chip.

### 2.5. Control System

The main control panel is used to control the camera, the light sources, the TEC module, and the data analysis system. The main control panel periodically turns on the light source for fluorescence excitation. The camera takes one picture of each fluorescent channel at the extension stage of each cycle. To protect the equipment, the real-time voltage of the light source is monitored. If the voltage becomes abnormal during the excitation period, the control panel would turn off the light source immediately.

The temperature of the TEC is controlled by the polarity of supplied power [34]. To extend the life of the TEC, The TEC controller will cut off the power supply when the temperature decreases to the threshold temperature of 55 °C. The controller also adjusts the fan speed according to the temperature to release heat in time and maintain a stable temperature.

The closed-loop PID controller is used to control the thermocycling. If the temperature difference between the TEC and the target is large, the TEC will run at full power to approach the target value as soon as possible. If temperature difference between the TEC and the target becomes relatively small, the PID controller will turn on to avoid temperature overshoot.

## 3. Results and Discussion

### 3.1. Design and Configuration of the Instrument

**Figure 3:**
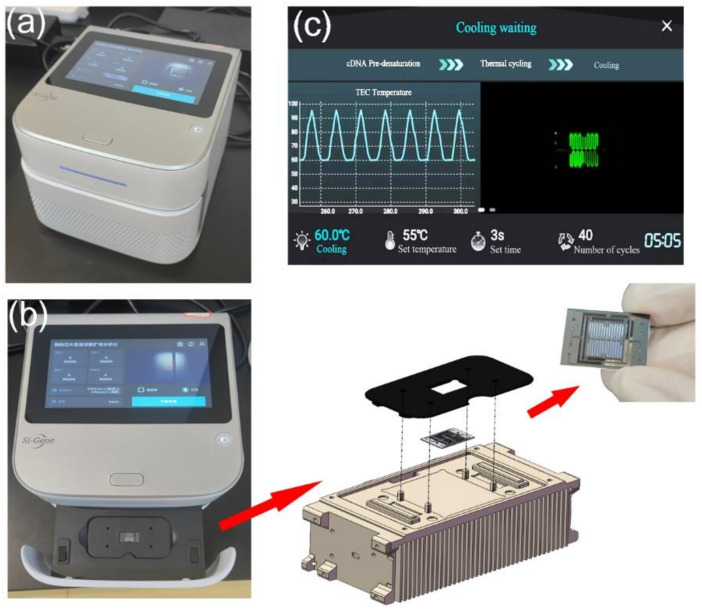
(**a**) Integrated ultrafast PCR detection equipment. (**b**) Slide mechanism for replacing the microfluidic chip. The miniaturized microfluidic chip is fixed on the thermocycling module through a holder. There are four positioning columns on the thermocycling module, which can precisely limit the position of the holder and ensure that the chip is on the best heating area. Two magnets are installed under the holder to ensure the stability of chip fixation through magnetic force. (**c**) The microcomputer can be operated through the touch screen.

All modules have been integrated into a compact and portable box for detection. The PCR detection equipment includes TEC thermocycling module, optical detection module, mechanical module for chip loading and external structure. The chip is fixed on a slide rail by a magnetic holder, as shown in Figure 3a,b. Below the chip is the TEC module. The optical detection system is directly above the chip, as shown in Figure 2. All modules of the equipment are packaged in an outer shell. A touch-screen microcomputer is equipped on the equipment, as shown in Figure 3c. The dimensions of the equipment are 228 mm × 205 mm × 151 mm (length × width × height), which can be easily carried and operated by one person.

### 3.2. Thermal Performance

**Figure 4:**
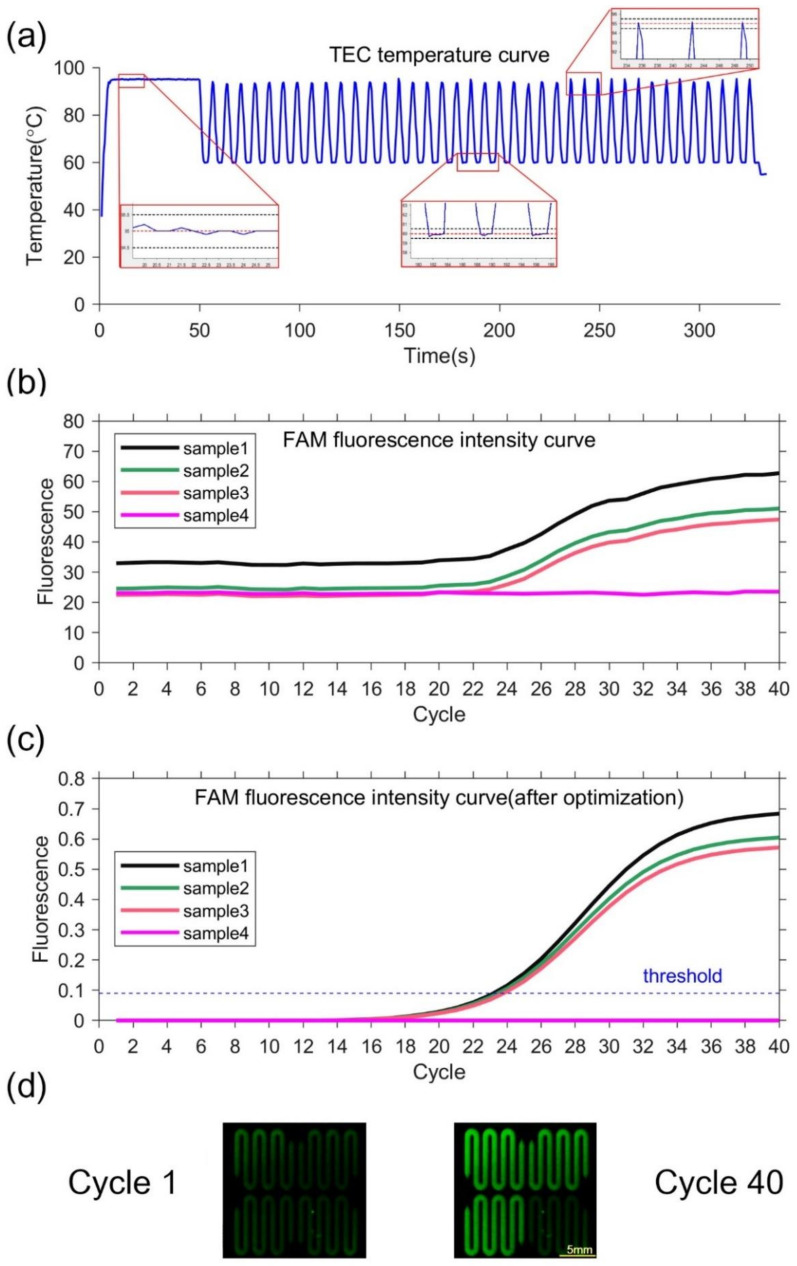
(**a**)TEC temperature curve and partial enlarged drawing. In the partial enlarged drawing, the red dotted line represents the set temperature, and the black dotted line represents the set temperature ±0.5 °C. (**b**)FAM fluorescence intensity curve. (**c**) FAM fluorescence intensity curve optimized by k-means clustering. The threshold is 0.09, so the Ct value is 23.222, 23.504 and 23.824. (**d**) Fluorescence image. The left figure is the first cycle, and the right figure is the 40th cycle.

The equipment can rapidly complete the temperature cycle with precise control. The temperature of the silicon-based microfluidic chip can change from 50 °C to 90 °C within 2 s. The average and maximum heating rate from 50 °C to 90 °C is 19 °C/s and 27 °C/s, respectively. The average and maximum cooling rate from 90 °C to 50 °C is 16 °C/s and 21 °C/s, respectively. The measured temperature deviation at 55 °C, 70 °C, and 95 °C is 0.2 °C, 0.1 °C, and 0.1 °C, respectively. No temperature overshoot occurs at the setting temperature point because of the PID control, as shown in Figure 4a. The temperature deviation and overshoot of the system is within ±0.2 °C and less than 0.2 °C, respectively. The data indicates that the equipment can control the temperature precisely.

### 3.3. Optical Performance

Fluorescence intensity is recorded to determine the repeatability and precision of the equipment. High (1.33 μmol/L), medium (0.889 μmol/L), and low (0.593 μmol/L) concentrations of calibration dyes are used to do repeated detection under different conditions, the CV (coefficient of variation) is less than 3%. The results prove that the on-chip fluorescence intensity detection has good repeatability. Randomly selected detection channels are tested under high, medium, and low concentration calibration dyes. The CV is less than 5%, which means a high precision. The fluorescence interference of different channels is tested. The results reveal that the background fluorescence intensity of other channels is smaller than the threshold of the target detection channel. High, medium, and low concentration nucleic acid samples are deployed for detection, the CVs of Ct values for FAM and ROX channels are less than 3% at three concentrations (Table A1).

### 3.4. Ultrafast PCR Detection

Negative and positive samples are used to verify the performance of the PCR equipment. Sample 1–3 with pseudovirus and Sample 4 as negative control are utilized. TEC temperature curve during the ultrafast PCR amplification procedure can be seen in Figure 4a. At each temperature set point, there is no temperature overshoot. The extension rate of the fast hot-start DNA polymerase that can reach 1000 bases per second [35]. The denaturation time can be set to 0 s and the annealing extension time to 2 s. Thus, our equipment can complete 40 cycles in 5 min. The fluorescence images show that the fluorescence intensities of samples 1–3 are significantly increased in 40 cycles, while the fluorescence intensity of sample 4 is unchanged, as shown in Figure 4d. The fluorescence intensity curves of samples 1, 2, and 3 have sigmoidal shape, indicating PCR amplification. In contrast, the negative control has little fluorescence change, as shown in Figure 4b,c. This demonstrate that our equipment is capable of ultrafast PCR. At the same time, the negative sample always keeps no fluorescence change, which can prove there is no cross interference between different chambers.

The ultrafast detection equipment is compatible with multiple PCR detection kits from different brands and can greatly reduce the total detection time. 10 different kits which take 1–1.5 h on standard PCR platform are tested. Test the same kits in the ultrafast PCR equipment only require 10–15 min, as shown in Table 1. The variable temperature parameters can be found in Table A2.

### 3.5. Comparison of the System’s Linearity

**Figure 5:**
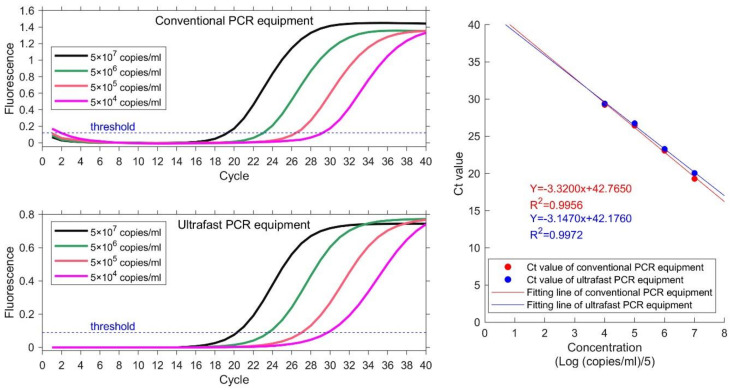
Results of four concentration gradient samples on conventional PCR equipment (HONGSHI) and ultrafast PCR equipment. The concentration from high to low is represented by four color curves of black, green, orange and magenta respectively. The Ct value and fitting line shows their correlative coefficients are 0.9956 and 0.9972 respectively.

Samples with 4 different concentrations are used to detect the sample linearity. Same experiment is carried out on conventional PCR equipment as reference. The nucleic acid samples are EBV plasmids from Quantitative Diagnostic Kit for EBV-DNA. The concentrations are 5 × 10^4^–5 × 10^7^ copies/mL. Ct value is obtained for each sample and plotted. Linear correlation coefficient R^2^ is calculated as the ratio of explained sum of squares (ESS) to total sum of squares (TSS). The results are illustrated in Figure 5 and Table 2. The instruction manual of this kit requires that the heating procedure is: 95 °C for 3 mins; 40 temperature cycles of 94 °C for 15 s and 60 °C. Both conventional PCR equipment and ultrafast PCR equipment set thermocycling parameters according to the requirements of the kit. The total running time of ultrafast PCR equipment is 40 min. The actual detection time of conventional PCR equipment is 71 min, because of its slow temperature varying rate.

The value of the linear regression coefficient R^2^ at each concentration is 0.9956 and 0.9972, which indicates good linearity between the detection results of each concentration. The linear regression coefficient calculated by ultrafast PCR equipment is higher than that of conventional PCR equipment. The results demonstrate that the equipment in this paper can acquire better results than conventional PCR equipment and can save a lot of time.

## 4. Conclusions

In this study, an ultrafast microfluidic PCR equipment is demonstrated. Compared with conventional PCR equipment, the equipment in this research is more compact and portable. The size of the equipment is only 228 mm × 205 mm × 151 mm (length × width × height). And the instrument manufacturing cost of the equipment is lower. The silicon-based microfluidic chip with thermal and fluid design can be heated evenly and avoid bubbles when adding samples through. TEC and silicon-based microfluidic chip are assembled to complete 40 PCR cycles within 5 min. In this paper, the probe method kit is used for detection with higher specificity. And the detection time is shortened from 1–1.5 h to only 10–15 min for all 14 PCR detection kits tested. The linearity of Ct value at different concentrations is 0.9972, which indicates good reliability of the measured results. The ultrafast PCR equipment can fulfill the tremendous public expectation for rapid detection on virus. The equipment tends to be suitable for rapid detection in different occasions. Future upgrade of the equipment is on the way to further enhance test efficiency and decrease production cost. Mass production can reduce the cost of silicon based chips. Finally, the cost of single inspection is reduced to an acceptable level.

## Figures and Tables

**Table 1:** Accelerating effect of PCR detection kits.

Brand	Kit	Total Time (Conventional)	Total Time (Ultrafast)
BoJie	2019-nCoV	1 h 13 min	9 min
ShuoShi		59 min	9 min
MoLe		1 h 19 min	8 min 38 s
ZhiJiang	HBV	1 h 22 min	9 min 30 s
	Mycoplasma pneumoniae and chlamydia pneumoniae	1 h 22 min	8 min 30 s
HuaFeng	Tgo	56 min 38 s	8 min 38 s
ZiJian	Influenza A + B virus	1 h 14 min	15 min
	DFV	1 h 14 min	8 min 32 s
	DFV-I	1 h 14 min	15 min
	DFV-II	1 h 14 min	14 min 35 s
	DFV-III	1 h 14 min	14 min 34 s
	DFV-IV	1 h 14 min	14 min 35 s
	Chikungunya virus	1 h 14 min	8 min 34 s

**Table 2:** Ct value of four concentration gradient samples.

Concentration	Ct Value	
	Conventional PCR Equipment	Ultrafast PCR Equipment
5 × 10^7^ copies/mL	19.29	20.05
5 × 10^6^ copies/mL	23.06	23.29
5 × 10^5^ copies/mL	26.44	26.74
5 × 10^4^ copies/mL	29.23	29.39

## Data Availability

All data are presented in the main text of this manuscript.

## Appendix A

**Table A1:** Fluorescence test.

Inspection Items	Standard Requirements	Test Conditions	Working Condition	Result
Fluorescence intensity detection	Repeat the test with high, medium and low concentration calibration dye, and the coefficient of variation (CV, %) shall not be greater than 3%.	High Concentration Dye (1.33 μmol/L)	Initial test	0.0 (+) %
			Rated working low temperature test, AC198V	0.0 (+) %
			Low temperature storage test	0.0 (+) %
			Rated working high temperature test, AV242V	1%
			Operation test, AV242V	1%
			High temperature storage test	0.0 (+) %
			Rated working damp heat test	1%
			Damp heat storage test	0.0 (+) %
			Vibration test	1%
			Impact test	1%
		Medium concentration dye (0.889 μmol/L)	Initial test	0.0 (+) %
		Low Concentration Dye (0.593 μmol/L)	Initial test	3 (−) %
	Randomly select m (m ≤ 4) detection holes and use medium and high concentration calibration dyes for detection. The coefficient of variation (CV, %) shall not be greater than 5%.	High Concentration Dye (1.33 μmol/L)		0.0 (+) %
		Medium concentration dye (0.889 μmol/L)		0.0 (+) %
		Low Concentration Dye (0.593 μmol/L)		0.0 (+) %
Fluorescence interference of different channels	The background fluorescence intensity of other channels is not higher than the fluorescence threshold of the current target detection channel.	FAM channel fluorescence threshold: 25.77		0.00
		ROX channel fluorescence threshold: 26.55		2.23
Sample detection repeatability	The CV of Ct value shall not be greater than 3% for the detection of high, medium and low concentration nucleic acid samples.	High Concentration Dye (1.0 × 10^8^ copies/mL)	FAM	2%
			ROX	2%
		Medium concentration dye (2.0 × 10^7^ copies/mL)	FAM	2%
			ROX	1%
		Low Concentration Dye (4.0 × 10^6^ copies/mL)	FAM	2%
			ROX	1%

**Table A2:** Variable temperature parameters and results of accelerated test of PCR detection kits.

Kit		Conventional PCR Equipment				Ultrafast PCR Equipment			
Brand	Detection Target	Number of Cycles	Temperature (°C)	Time	Total Time	Total Time	Time	Temperature (°C)	Number of Cycles
BoJie	2019-nCoV		95	5 min	1 h 13 min	9 min	1 min	95	
		40	95	10 s			1 s	95	40
			55	40 s			5 s	55	
ShuoShi			97	1 min	59 min	9 min	1 min	95	
		40	97	5 s			1 s	95	40
			58	30 s			5 s	55	
MoLe			95	30 s	1 h 19 min	8 min 38 s	1 min	95	
		40	94	10 s			1 s	94	40
			58	60 s			5 s	58	
			58	60 s			5 s	58	
ZhiJiang	HBV		37	2 min	1 h 22 min	9 min 30 s	2 min	94	
			94	2 min					
		40	93	15 s			1 s	93	40
			60	60 s			5 s	60	
	Mycoplasma pneumoniae and chlamydia pneumoniae		94	2 min	1 h 22 min	8 min 30 s	45 s	94	
		40	93	15 s			1 s	93	40
			60	60 s			5 s	60	
HuaFeng	Tgo		94	5 min	56 min 38 s	8 min 38 s	45 s	95	
		40	95	5 s			1 s	95	40
			60	25 s			5 s	60	
ZiJian	Influenza A + B virus		95	10 min	1 h 14 min	15 min	60 s	95	
		40	95	5 s			5 s	95	40
			60	45 s			10 s	60	
	DFV		95	10 min	1 h 14 min	8 min 32 s	60 s	95	
		40	95	5 s			1 s	95	40
			60	45 s			5 s	60	
	DFV-I		95	10 min	1 h 14 min	15 min	60 s	95	
		40	95	5 s			5 s	95	40
			60	45 s			10 s	60	
	DFV-II		95	10 min	1 h 14 min	14 min 35 s	60 s	95	
		40	95	5 s			5 s	95	40
			60	45 s			10 s	60	
	DFV-III		95	10 min	1 h 14 min	14 min 34 s	60 s	95	
		40	95	5 s			5 s	95	40
			60	45 s			10 s	60	
	DFV-IV		95	10 min	1 h 14 min	14 min 35 s	60 s	95	
		40	95	5 s			5 s	95	40
			60	45 s			10 s	60	
	Chikungunya virus		95	10 min	1 h 14 min	8 min 34 s	60 s	95	
		40	95	5 s			1 s	95	40
			60	45 s			5 s	60	
